# Serum leptin levels decrease after permanent MCAo in the rat and remain unaffected by delayed hyperbaric oxygen therapy

**DOI:** 10.1186/2045-9912-3-8

**Published:** 2013-03-19

**Authors:** Jun Mu, Robert P Ostrowski, Paul R Krafft, Jiping Tang, John H Zhang

**Affiliations:** 1 Department of Neurology, The First Affiliated Hospital of Chongqing Medical University, Chongqing, China; 2 Department of Physiology, Loma Linda University School of Medicine, Loma Linda, CA, China; 3 Department of Neurosurgery, Loma Linda University School of Medicine, Loma Linda, CA, China; 4 Department of Experimental and Clinical Neuropathology, Medical Research Centre, Polish Academy of Sciences, Warsaw, Poland; 5 Department of Physiology, Loma Linda University School of Medicine, Risley Hall, Room 223, Loma Linda, CA, 92354, China

**Keywords:** Hyperbaric oxygen therapy, Leptin, Neuroprotection, MCAo, Rats

## Abstract

Hyperbaric oxygen therapy (HBOT), referring to the medical use of oxygen at a level higher than atmospheric pressure, exerts neuroprotective effects after ischemic stroke via various mechanisms. It has been demonstrated that HBOT modulates the synthesis and degradation of hormones. Leptin, an adipose derived hormone, has been found to confer neuroprotection following experimental stroke. However, it is not known whether HBOT alters leptin concentrations after permanent middle cerebral artery occlusion (pMCAo) in the rat. In this present study, we aimed to investigate the effect of HBOT on the serum concentration of leptin in rats subjected to pMCAo. HBOT was initiated 48 hrs after experimental pMCAo, at 2.5 atmospheres absolutes with 100% oxygen, 1 hr a day for 10 consecutive days. Body weight, neurobehavioral deficits and infarct size were evaluated. Blood was collected on day 1 and day 16 following HBOT. Serum leptin concentrations were measured with ELISA. Delayed HBOT reduced infarct size and improved neurobehavioral scores. Decreased serum levels of leptin were found in treated and untreated pMCAo animals, compared to the sham group on day 1 (*P* > 0.05) and day 16 (*P* < 0.05). However, no statistical significance was found between HBOT and the air group. We concluded that the neuroprotective effects of delayed HBOT in pMCAo rats were unlikely to be exerted through changes in the serum concentration of leptin.

## Introduction

Hyperbaric oxygen therapy (HBOT) refers to the medical use of oxygen at levels higher than atmospheric pressure. The potential applications of HBOT following ischemic stroke have been investigated in various animal models and clinical trials [1-5]. Previous studies utilizing experimental models of ischemic stroke demonstrated that HBOT improved neurological functions [3], reduced infarct size [3,4], decreased hemorrhagic transformation [5], and promoted neurogenesis [6-9]. It has also been suggested that these effects are achieved through a variety of mechanisms, including reduction of oxidative and metabolic stress, amelioration of brain edema, suppression of inflammation and apoptosis, as well as stabilization of the blood brain barrier, by activation of cellular transcription factors [10]. A major effect of HBOT is the elevation of oxygen partial pressure within the blood and therefore the restoration of oxygen supply to the penumbra after ischemic stroke [11].

Recent evidence revealed that HBOT modulates the synthesis and degradation of several hormones. Edstrom and his colleagues investigated the effect of HBOT on endocrine organs and found that hyperbaric oxygen evoked adrenal hypertrophy, reduced thymus weight but increased thyroid weight [12]. Animal experiments further demonstrated that serum levels of adrenal epinephrine and norepinephrine increased in spontaneously hypertensive rats that received HBOT (2 ATA, 90 min) [13]. The serum concentration of cortisol, as well as the concentration of glucocorticoid receptors in the rat’s lung were reduced following HBOT (3 ATA, 5 hrs) [14]. In clinical studies, HBOT (2.0 ATA, 10 days) increased serum levels of estrogen and estrogen receptors in infertile patients [15], and decreased erythropoietin (EPO) concentrations in healthy subjects (2.5 ATA, 90 min) [16]. HBOT (2.5ATA, 1 hr X 10 sessions) improved the metabolic control and reduced insulin requirements in patients with type 2 diabetes mellitus (T2DM) after stem cell transplantation [17]. However, short-term HBOT (2.5 ATA, 90 min X 3 days) did not alter levels of circulating insulin, insulin-like growth factor (IGF), leptin, interleukin-8 (IL-8) or nitric oxide (NO) in patients with T2DM [18]. Furthermore, single HBOT (2.5 ATA, 1 hr) failed to induce a generalized hormonal stress reaction in 8 divers, who showed normal serum concentrations of epinephrine, norepinephrine, antidiuretic hormone (ADH), atrial natriuretic peptide (ANP) and renin after the HBOT session [19]. In summary, while it has been suggested that HBOT interrelates with endogenous hormone pathways, long-term HBOT, rather than single or short-term based therapies, affected the hormone balance to a greater extend. So far, there is no research related to hormone profiles induced by HBOT in ischemia patients or animal models. Thus, it is not clear whether HBOT can ameliorate ischemic brain injury by modulating the serum levels of specific hormones.

Among all hormones modulated with HBOT, leptin is a promising candidate for the treatment of ischemic stroke [20]. Leptin, a 16 kDa peptide produced and released by adipocytes, acts via its receptors in the hypothalamus to decrease appetite and increase energy expenditure [21,22]. The secretion of leptin from adipocytes is regulated by multiple factors, including body weight, food intake, insulin and hypoxia [23]. Exogenous administration of leptin, attenuated neuronal hypoxic injury in both in vivo [24,25] and in vitro [20,26] studies. Endogenous leptin expression as well as mRNA levels were found increased in the peri-infarct brain tissue up to 24 hrs after permanent middle cerebral artery occlusion (pMCAo) in mice [27]. In oxygen-breathing mice, serum leptin was increased six to seven fold by hyperoxia and leptin mRNA was elevated within the adipose tissue [28]. To date, no studies have investigated the relationship between HBOT and endogenous leptin in ischemic stroke models. In this present study, we hypothesized that multiple and long term HBOT can exert neuroprotection by increasing the serum level of leptin in pMCAo rats.

## Materials and Methods

### Animals

All experiments were approved by the Institutional Animal Care and Use Committee (IACUC) of Loma Linda University. Seventy-two Sprague–Dawley male rats (Harlan, Indianapolis, Ind) weighing 240 to 270 g were randomly divided into the following groups: sham-operated + air (n = 20), air (pMCAo + air) (n = 28) and HBOT (pMCAo + 2.5 ATA HBOT) (n = 24). Animals were fed with a standard laboratory diet, and were housed in a temperature-controlled room (24°C), illuminated for 12 hrs daily (lights on from 5 AM to 5 PM).

### Surgery

Rats were anesthetized via intraperitoneal injection of ketamine (100 mg/kg) and xylazine (8 mg/kg). The rectal temperature was monitored and kept at 37.0 ± 0.5°C, by using a feedback-regulated heating system during surgery. Permanent focal ischemia was induced by occluding the right middle cerebral artery (MCA) via the intraluminal technique [29]. Briefly, a 4–0 nylon monofilament suture with a slightly enlarged round tip was inserted into the stump of the external carotid artery (ECA) and advanced into the lumen of the internal carotid artery (ICA) until it reached and occluded the MCA. The distance from bifurcation of the common carotid artery (CCA) to the tip of the suture averages 18–20 mm. Sham operated animals were subjected to the same surgery procedure, except that no suture was inserted.

### HBOT paradigm

**Figure 1:**
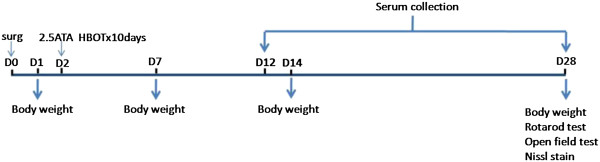
Experimental design.

HBOT was initiated 48 hrs after the pMCAo, at 2.5 atmospheres absolutes with 100% oxygen, 1 hr a day for 10 consecutive days (Figure 1). Rats were pressurized in a research hyperbaric chamber (1300B, Sechrist) with an oxygen flow of 22 L/min. Compression and decompression was maintained at a rate of 5 psi (pounds per square inch)/min. For sham and vehicle groups, normobaric air was used.

### Neurobehavioral Testing

At 4 weeks after pMCAo, rotarod and open field tests were performed to assess motor coordination, motor learning and locomotor activity respectively. The rotarod test consists of a rotating horizontal cylinder divided into 9.5 cm-wide lanes. When animals were placed onto it, they must move forward continuously to avoid falling off. Latency to fall off is recorded by a photobeam circuit [30]. In the open field tests, animals were observed for 30 min in opaque open-topped plastic boxes (49 cm X 35.5 cm X 44.5 cm) and recorded by an overhead camera. The animals' movements were analyzed by the computerized tracking system [30].

### Body weight

All rats were weighed before surgery and on days 1, 7, 14 and 28 after surgery. Body weight changes were expressed as the percentage of the baseline value obtained prior to surgery.

### Nissl staining

On day 28 after surgery, rats were transcardially perfused with 200 mL of ice cold phosphate-buffered saline (PBS), followed by 300 mL of phosphate-buffered 10% formalin. Brains were postfixed and cryoprotected as described [31]. Detection of infarct tissue was performed on 10 consecutive sections cut into 10 μm-thick coronal sections at 1 mm interval (from Bregma +3 to Bregma −7). For Nissl staining, the sections were dried, rehydrated and immersed in 0.5% cresyl violet for 2 minutes [21]. After washing in water, sections were dehydrated in graded alcohols, cleared in xylene and cover-slipped with Permount (Thermo Fisher Scientific). Infarction volume was quantitatively analyzed by sum of the infarction area in all brain slices,multiplied by thickness of each slice.

### Serum leptin measurement

Serum leptin concentration in normal rats exhibits a diurnal pattern, peaking at 2 hrs after dark [22]. In view of this, blood was collected between 7:00 PM to 9:00 PM at 1 and 16 days after the last HBOT session. Serum was separated by centrifugation at 3,000 g for 10 minutes at 4°C and stored at −20°C. Serum leptin concentrations were measured with a rat leptin enzyme-linked immunosorbent assay (ELISA) kit (Millipore, MA, USA).

### Statistical analysis

Results were expressed as mean ± SEM. Intergroup differences for were analyzed by ANOVA, using SigmaStat (Systat Software, CA, USA). A P value of 0.05 or less was considered significant.

## Results

### Mortality rate, body weight, neurobehavioral data and infarct volume

The mortality at day 28 was 0% (0/20) in the sham group, 28.6% (8/28) in the air group (pMCAo + air) and 8.3% (2/24) in the HBOT group. The mortality in the HBOT group tended to be lower than that of the air group (*P* > 0.05).

**Figure 2:**
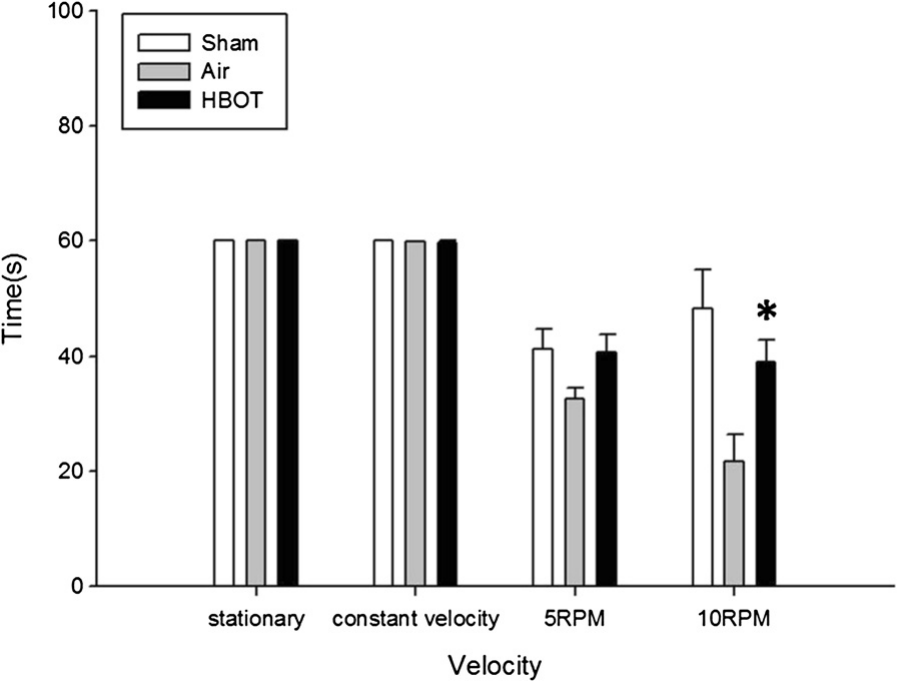
**Effects of HBOT on Rotarod test.** *P < 0.05 vs air group. The animal number was 20, 20 and 22, respectively in the sham, air and HBOT group.

**Figure 3:**
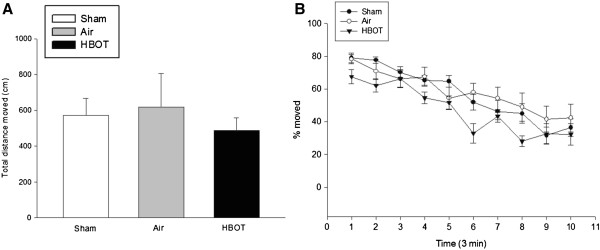
**Effects of HBOT on open field test.** There was no difference between air and HBOT group in the total distance moved (cm) (Figure 3**A**) and total duration of moving (%) (Figure 3**B**). The animal number was 20, 20 and 22, respectively in the sham, air and HBOT group.

Rotarod test results of motor coordination and motor learning were significantly improved on 10 RPM compared with the air group (*P* < 0.05) (Figure 2). Locomotor activity was evaluated by open field test, expressed as total distance moved (Figure 3A) and as total duration of moving (Figure 3B). However, no statistical significance was noted between the air and HBOT groups (*P* > 0.05).

**Figure 4:**
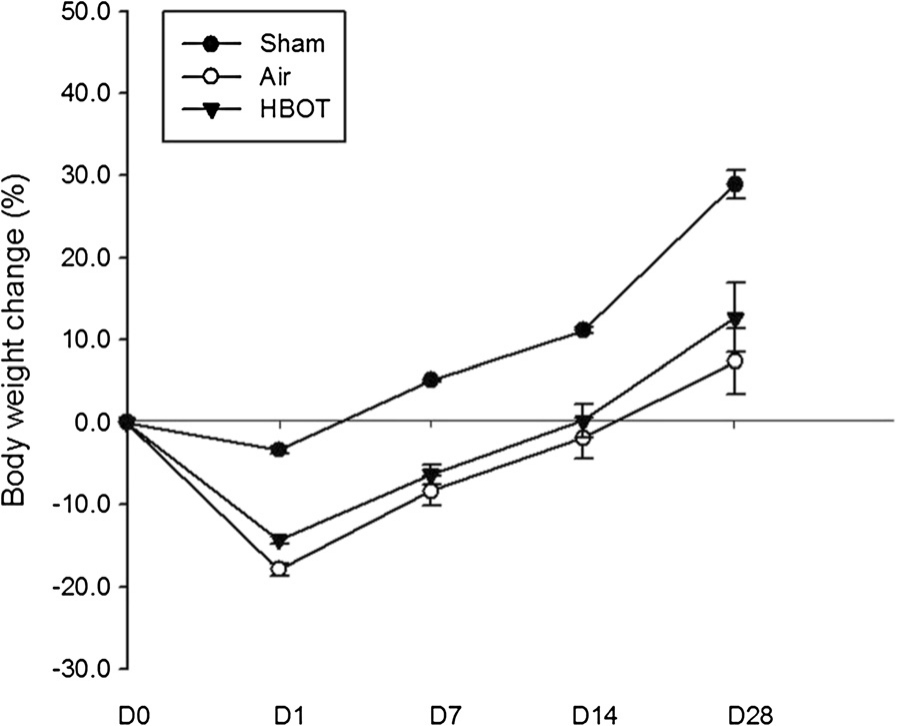
**Weight changes on day 1, 7, 14 and 28.** Body weight of rats expressed as percent difference of preoperative weight. There were no statistically significant differences in the baseline weight between the groups. There were no statistically significant differences in weight between the HBOT group as compared to air group at each time point. The animal number was 20, 20 and 22, respectively in the sham, air and HBOT group.

Body weight was recorded on day 1, 7, 14 and 28 (Figure 4) and was expressed as percent difference of preoperative weight. There was no statistically significant differences in the baseline weight between the groups. There was no statistically significant differences in weight between the HBOT group as compared to air group at each time point (*P* > 0.05).

**Figure 5:**
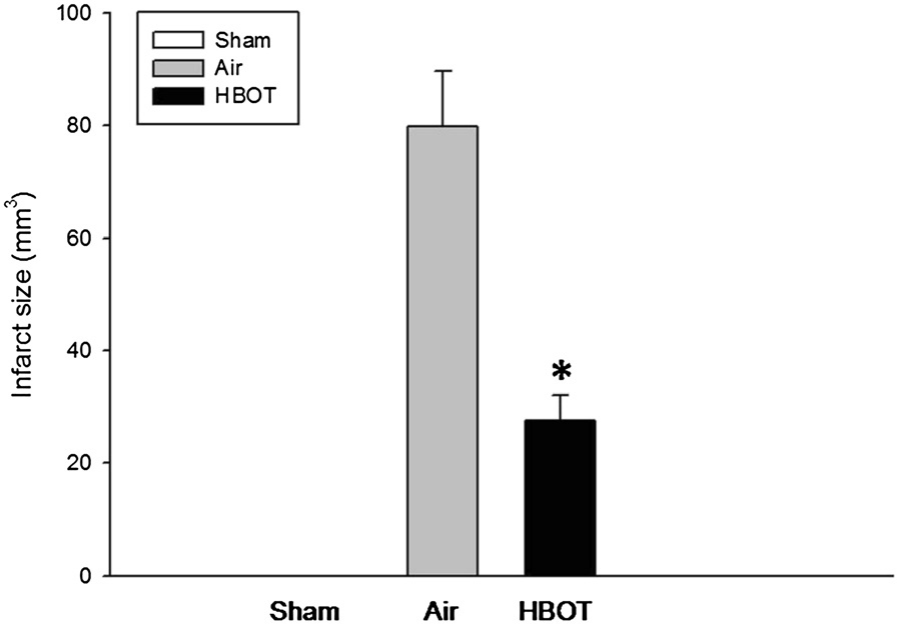
**Changes of infarct volume at 28 days after HBOT.** The infarct volume was calculated in mm^3^ of infarcted tissue per ipsilateral hemisphere. *P < 0.05 vs air group. The animal number was 20, 20 and 22, respectively in the sham, air and HBOT group.

Infarct volume was reduced in the HBOT group compared with the air group on day 28 after surgery (*P* < 0.05) (Figure 5).

### Leptin levels

**Figure 6:**
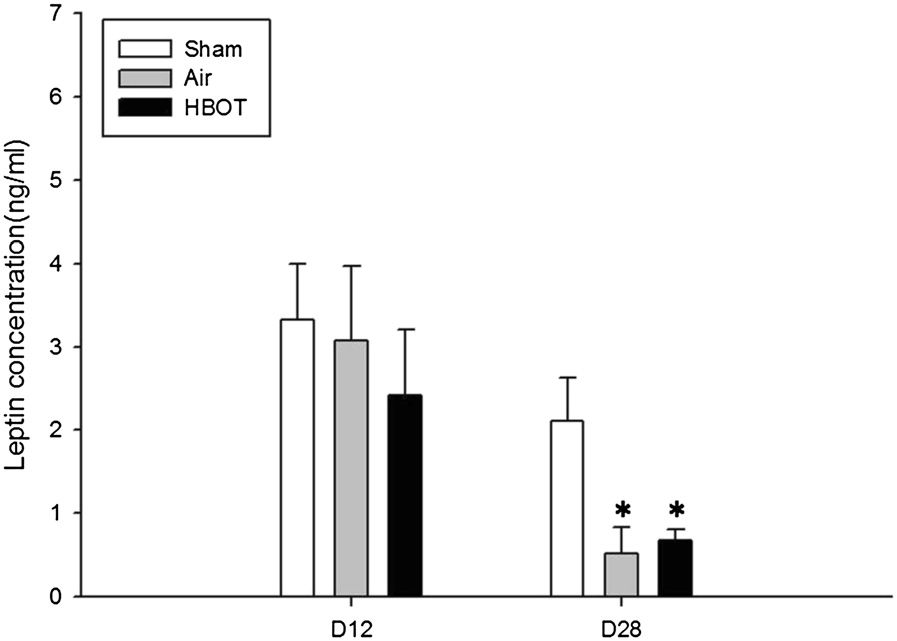
**Changes of leptin concentration.** Leptin level in the serum of treated animals showed no statistically significant difference compared with that in the serum of air rats both on day 1 and day 16 after last session of HBOT. *P < 0.05 vs sham group. The animal number was 20, 20 and 22, respectively in the sham, air and HBOT group.

Although leptin level tended to be lower in the pMCAo groups compared with sham operated animals, there was no statistically significant difference between these two groups (pMCAo + air vs. pMCAo + HBOT) 1 day after the last session of HBOT. On day 16 after the last session of HBOT, serum level of leptin was found further reduced in the pMCAo groups compared with the sham group (Figure 6) (*P* < 0.05). The leptin concentration in the serum of treated animals showed no significance compared with that in the serum of air-treated rats (*P* > 0.05).

## Discussion

In this present study, we applied delayed HBOT to pMCAo rats in order to explore whether the treatment exerts neuroprotection by modulating the serum concentration of leptin. We found that daily HBOT, initiated 48 hrs after pMCAo, reduced infarct size and improved neurological scores. Both air treated and HBOT pMCAo groups showed a tendency toward decreased leptin levels compared to sham animals on day 1 (*P* > 0.05) and a significant decrease leptin level on day 16 (*P* < 0.05), measured after the last session of HBOT. The leptin level in the HBOT group tended to be higher than the air group without any noted statistical significance.

PMCAo model, compared to the transient MCAo model, was selected for the present study as it represents the natural evolution of focal ischemia in clinical situations without spontaneous recanalization. While both models are well reproducible, pMCAo model was less variable in terms of lesion size [32]. Also, it is difficult to start the HBOT immediately from the very acute stage in ischemic stroke patients, for the reason of recombinant tissue plasminogen activator (rt-PA) administration, intensive care monitoring and generally unavailability of spacious HBO chamber. Therefore, the latest initiation of HBOT was applied 24 hrs after the transient MCAo [3]. As demonstrated already in our previous study, delayed and continuous HBOT (starting 48 hrs after ischemia) effectively reduced infarct size and improved neurobehavioral scores.

It has been known that serum leptin concentration in normal rats exhibits a diurnal pattern and peaking at 2 hrs after dark [22]. The light dark cycle begins from 5:00 AM to 5:00 PM in our study. As a result, serum was uniformly collected from 7PM to 9PM. Since HBOT sessions lasted for 10 days, 2 time points were chosen for blood collection: one day after the last session of HBOT for the acute effect of HBOT on level of leptin and 16 days after the last session of HBOT for the observation of chronic effects.

In this study, no significant differences of body weight between air group and HBOT group were found at each time point. Animals subjected to pMCAo showed significantly more weight loss compared with the sham group. That partly explained why 1 day after the last session of HBOT, there was a decrease of leptin level in both air and HBOT group. However, more than 2 weeks later, when body weight recovered to above the preoperative level, there was a further decrease of leptin level in both groups, suggesting the change of leptin level was irrelevant to that of body weight. Although the leptin level in HBOT group tended to be higher than that of the air group, the difference was not statistically significant. Clinical investigations revealed that acute stroke patients have increased serum levels of leptin [33]. It has been reported that increased level of leptin is associated with abdominal obesity [34], higher risk of ischemic stroke [33] and post-stroke depression [35]. Exogenous administration of leptin, exerted neuroprotection against ischemia brain injury both in vivo [24,25] and in vitro [20,26]. Barazzone-Argiroffo and collegues reported that serum leptin was increased six to seven fold in oxygen-breathing hyperoxia mice [28]. It is postulated that acute hypoxia-induced increase in leptin level produces neuroprotection and HBOT may further increase the leptin level therefore enhance neuroprotection. However, we didn’t observe any leptin increase either 1 day or 16 days after last session of HBOT. Possible explanations may include: 1) leptin level increased within the first few days of ischemia then felled at day 12; 2) the HBOT-induced increase in the level of leptin was absorbed by the ischemia insult to cause a neuroprotective effect of reducing the infarct size and improving the neurobehavioral scores; 3) HBOT sessions were not long enough to cause further increase of leptin, because on day 28, there was slightly higher level of leptin in HBOT group, compared with the air group; 4) serum level of leptin is not sensitive enough to be detected as compared to the brain tissue leptin expression and mRNA level.

To further evaluate whether HBOT modulates the level of leptin, more groups at different time points should be considered: 1) HBOT sessions further extended; and 2) HBOT initiated immediately after ischemia. Aside from the serum level of leptin, gastric and hypothalamic leptin level might be tested at the same time. In addition, leptin mRNA and leptin receptors are important index modulated by HBOT.

## Conclusions

Delayed HBOT produced neuroprotective effects on pMCAo rats. Serum leptin levels were decreased after pMCAo in rats and remained unaffected by delayed HBOT. The decreased level of serum leptin in HBOT group was possibly because of counteracting effect of ischemia insult against HBOT. Noticeably though, the leptin level in the HBOT group showed a tendency to increase, compared to air group. To further elucidate whether HBOT exerts the neuroprotection through modulation of leptin expression, leptin level, mRNA and receptors need to be examined at various additional time points, as outlined above.

## Competing interests

The authors report no conflict of interests.

## Authors’ contributions

JM conducted animal surgery and wrote the first draft of this paper. RPO provided guidance for surgery and technical support. PRK conducted animal behavioral testing and assisted in molecular biological studies. JT and JHZ provided guidance during study design, molecular biological studies, and provided overall supervision. All authors read and approved the final manuscript.
